# Guided (VENTRI-GUIDE) versus freehand ventriculostomy: study protocol for a randomized controlled trial

**DOI:** 10.1186/1745-6215-15-478

**Published:** 2014-12-05

**Authors:** Asita Sarrafzadeh, Nicolas Smoll, Karl Schaller

**Affiliations:** Division of Neurosurgery, Geneva University Hospitals, Geneva Neuroscience Center, Faculty of Medicine University of Geneva, Rue Gabrielle-Perret-Gentil 4, CH-1211 Genève 14, Switzerland; Department of Surgery, Frankston Hospital, Hastings Road Frankston, Victoria, Melbourne 3199 Australia

## Abstract

**Background:**

Despite the widespread use of external ventricular drainage, revision rates, and associated complications are reported between 10 and 40%. Current available image-guided techniques using stereotaxy, endoscopy, or ultrasound for catheter placements remain time-consuming techniques. Recently, a smartphone-assisted guide with high precision has been described. The development of an easy-to-use, portable, image-guided system could reduce the need for multiple passes and improve the rate of accurate catheter placement. This study aims to prospectively compare in a randomized controlled manner the accuracy of the freehand pass technique versus an easy-to-use, portable, adjustable guiding device for ventriculostomy catheter placement.

**Methods/Design:**

This is a single center, prospective, randomized trial with a blinded endpoint (ventricular catheter tip location) assessment. Adult patients with the indication for ventriculostomy, as proven by computed tomography (CT), will be randomly assigned to the treatment group or the control group. For patients in the treatment group, ventriculostomy will be performed using an adjustable guiding device and DICOM (Digital Imaging and Communications in Medicine) image-reading software assistance (for example, using a mini-tablet) based on preoperative CT imaging.

Patients in the control group will receive standard freehand ventriculostomy using anatomical landmarks. The catheter may be placed for external drainage or internal (ventriculoperitoneal) shunting in both groups. The primary outcome measure is the rate of correct placements of the ventricular catheter, defined as a score of 1 to 3 on grading system for catheter tip location on a postoperative CT scan. Participants will be followed for the duration of hospital stay, an expected average of two weeks. The primary outcome will be determined by one of the authors blinded to the treatment allocation. We aim to include 236 patients in three years. Secondary outcome measures include: frequency of placements required, frequency of completed placements within the ventricle of the perforated part of the catheter tip, frequency of very early and early shunt failures (revision of the ventricular drainage within 24 hours and within the hospital stay), frequency and percentage of complications (procedure-related and nonsurgical) at discharge.

**Discussion:**

This is the study design of a single center, prospective, randomized controlled trial to investigate whether guided ventriculostomy is superior to the standard freehand technique. One strength of this study is the prospective, randomized, interventional type of study testing a new easy-to-handle guided versus freehand ventricular catheter placement. A second strength of this study is that the power calculation is based on catheter accuracy using an available grading system for catheter tip location, and is calculated with the use of recent study results of our own population, supported by data from prominent studies.

**Trial registration:**

Clinicaltrials.gov identifier: NCT02048553 (registered on 28 January 2014).

## Background

Ventriculostomy is a common procedure in neurosurgery and accurate placement of the ventricular catheter is one of the most important variables in the longevity of shunt survival [1, 2]. Despite this large volume of patients and technological advances, failure rates due to proximal catheter obstruction with the freehand technique remain high (12%, 32%,36%, 38%, and 45% in studies [3–7], respectively. Optimal placement in the ipsilateral anterior horn of the lateral ventricle just anterior to the foramen of Monro depends on accurate placement of the catheter tip in the ventricle, away from the choroid plexus and injured ependyma [8], and on proper insertion trajectory and catheter length [9]. In a retrospective study in 90 patients using surface anatomical landmarks alone, only 56% of the catheter tips were in the ipsilateral lateral ventricle, 7% were in the contralateral lateral ventricle, 8% were in the third ventricle, 6% were within the interhemispheric fissure, and 22% were within extraventricular spaces [10].

The high rate of malplacements led to a search for improved methods that might result in significant patient benefits. In the last 25 years, techniques using a guide [11], calculating optimal catheter lengths combining computed tomography (CT) [12], and stereotactic coordinate-guided freehand methods based on ventricular landmarks [9, 13] have been developed. This was followed by the more sophisticated methods of the ultrasound transducer [14–16], neuronavigation, and endoscopic guidance [17].

A very recent study comparing the accuracy of ventricular catheter placement using the freehand technique, ultrasonic guidance, and stereotactic neuronavigation identified the use of the freehand technique as the only risk factor for inaccurate placement [7]. Current available image-guided techniques for catheter placements can be time consuming [14, 18], expensive, and may need a larger burr hole [19]. The development of an easy-to-use, portable, image-guided system could reduce the need for multiple passes and improve the rate of accurate catheter placement [16]. Recently, a smartphone-assisted guide was tested in 35 patients with no ventricular catheter failure occurring during the follow-up period [20].

As there is a need to improve current practices in the placement of ventricular catheters, this study aims to compare prospectively and in a randomized controlled manner the accuracy of the freehand pass technique versus a portable adjustable guiding device for ventriculostomy catheter placement.

## Methods/Design

This is a single center, prospective, randomized trial with a blinded endpoint (ventricular catheter tip location) assessment. Adult patients with the indication for ventriculostomy, as proven by CT, will be randomly assigned to the treatment group or the control group. For patients in the treatment group, a ventriculostomy will be performed using an adjustable guiding device and DICOM (Digital Imaging and Communications in Medicine) image-reading software assistance (for example, using a mini-tablet) based on preoperative CT imaging.

### Design and setting

**Figure 1:**
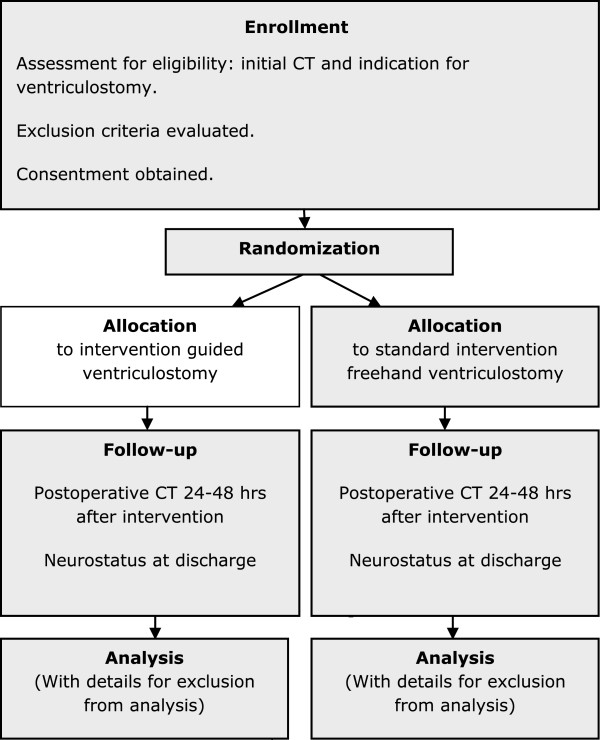
**Study design.**

The guided versus freehand ventriculostomy (VENTRI-GUIDE) study will be performed as a -two-arm, single center, randomized controlled trial to compare an intervention group using a guided placement of a ventricular catheter to a control group receiving standard (freehand) ventriculostomy with a blinded endpoint (catheter tip location) assessment (Figure 1).

The present study is in compliance with the Helsinki Declaration. The protocol of this study was approved by the Ethics Committee on Human Research of the Medical Faculty of the University of Geneva, Switzerland (reference number: CER 13–175). At the planned start of the study (1 February 2014), the study center will start randomization.

Adults with indication for a ventricular drainage are eligible for trial participation and are recruited for this study. The choice of the method of the respective operative procedure is via randomization. All other surgical and medical treatment is performed according to local guidelines and standard operating procedures.

### Subject inclusion criteria

The inclusion criteria for this study are patients aged 18 years or older with an indication for a ventricular drainage (such as hydrocephalus, slit ventricles, or pseudotumor cerebri).

### Subject exclusion criteria

Patients who meet any of the following criteria will be excluded from the study: pregnancy, concurrent participation in another interventional trial (participation in an observational trial is not an exclusion criteria), or if a frontal burr hole is not available.

#### Randomization

The randomization process will start as soon as possible after consent to the study has been obtained. Any patient meeting the inclusion criteria and not violating the exclusion criteria may participate in the VENTRI-GUIDE study and be randomized to either a guided or freehand ventriculostomy. Online randomization will be performed by the treating physician in the study center using permuted blocks, to ensure an equal number of patients in both study arms.

### Surgical technique for guided group intervention

The study is conducted in the neurosurgical department of a Swiss single center university hospital in adult patients with an indication for a ventricular drainage (such as hydrocephalus, slit ventricles, or pseudotumor cerebri). Data management and monitoring will be performed by the Geneva study center and statistical analysis by one of the authors (NS).

For guidance, an easy-to-handle instrument, assisted by DICOM image-reading software (for example, mini-I-pad™ application software; Apple™ Inc(Apple Inc., Cupertino, California, United States ), is used in order to achieve precise placement of ventricular catheters on an every-case basis. The catheter may be placed for external drainage or internal (ventriculoperitoneal) shunting.

For patients in the guided group, catheter placement is performed in the operating theatre during anesthesia under routine sterile conditions and antibiotic prophylaxis. The smartphone-assisted technique used for guided placing of a ventricular catheter through a burr hole has been previously described in detail [20]. The only difference in this study is the use of an easy-to-use portable DICOM image-reading software assistance (for example, using a mini-tablet) available for all neurosurgeons instead of a smartphone. In brief, the patient is positioned and draped in typical sterile fashion for a ventriculostomy (if necessary, the operation field is enlarged for shunt placement. Then, a frontal burr hole is made using a standard adult perforator.

The guiding instrument consists of a base with three pins to be rigidly placed on the bone surface over the burr hole. A semicircular guide rod (Aesculap AG, Tuttlingen, Germany is mounted to the base in which an angle scale is engraved. Within the guide rod, a tube can be individually adjusted in one orientation at different angulations with a range from -60° to 60°. Different tubes are designed with inner diameters of 2, 2.5, and 3 mm in order to guide catheters at different sizes. At the respective angle adjustment, the tube is fixed to the guide rod by a mounting screw. The base is opened at one side to enable the view or insertion of any instrument towards the lower opening of the tube. The guiding tool is positioned parallel to the midline along the engraved linear markings on the base of the instrument. Thereby, a rectangular insertion towards the sagittal convexity and an individual insertion angle towards the coronal tangent at the entry point can be established via the tube.

After opening the dura, once the appropriate trajectory is chosen, a ventricular catheter is inserted according to the pre-calculated insertion angle (for a detailed description see Thomale *et al*. [20]). After receiving tactile feedback from entering the ventricle, the stylet is removedCerebrospinal fluid (CSF) drainage ensures that the ventricular catheter is correctly in place. Catheters deemed to be suboptimally placed are repositioned when necessary. We rely on length markings on the ventricular catheter and, when possible, flow through the distal catheter to ensure the catheter has not been moved after fixation to the skin and is continuing to provide a conduit for CSF. The remainder of any following shunt procedure or connection to an Ommaya reservoir will be performed using standard techniques.

The classical freehand technique uses anatomical landmarks and the catheter is placed in the anterior horn of the lateral ventricle through a burr hole just anterior to the coronal suture in the mid-pupillary line (at the level of Kocher’s point; a point on the surface of the cranium 2.5 cm from the midline and 1 cm anterior to the coronal suture, approximately 11.5 cm above the nasion). The target is the ipsilateral frontal horn just anterior to the foramen of Monro to avoid the choroid plexus. The right nondominant side is preferred. The catheter is directed freehand, using external landmarks in the coronal plane towards the ipsilateral medial canthus and in the sagittal plane through the external auditory meatus. A post-procedural CT scan of the brain is performed within 24 hours as part of standard care for postoperative control, or earlier in case of postoperative CSF drainage problems.

### Mini-tablet application

Regular DICOM viewing software, commonly used in clinics to evaluate radiological imaging, integrates simple measurement tools (such as distance or angle measurements). This technique is used to define a trajectory with an entry point at the frontal paramedian convexity towards the foramen of Monro in a coronal section. Then, measurements can be taken to determine the angle between the trajectory and the respective tangent at the entry point on the skull surface. An iPhone™ (Apple Inc., Cupertino, California, United States) software application is used, importing anonymous imaging material. This image should be a coronal section with the lateral ventricles shown at the level of the anterior commissure. The next step is to define a paramedian entry point on the surface of the skull by tapping the finger on the respective spot. Then, the two feet are virtually placed on the skull surface. A rectangular trajectory orientation of the tube is then shown as a dotted line and can be shifted if necessary. The angle deviation from a rectangular insertion is then given by the software as a value. In addition, the finger may be placed on the target within the ventricle, and the length of the catheter will be given as distance value. As an alternative, the CT scan (at the level: coronal section with the lateral ventricles shown at the level of the anterior commissure) can be photographed and used for angle calculations.

### Outcome assessment

#### Primary outcome measures

The first primary outcome measure will be the frequency and percentage of correct placements of the ventricular catheter, assessed by the three-point scale of Kakarla *et al*., with an additional 1a/1b criteria (Table 1) [21]. The effect size will be measured using relative risk, absolute risk reduction, and numbers needed to treat. The second primary outcome measure will be the frequency and percentage of acute ventricular revisions (within one week for an improperly placed catheter). Assessment is performed by an investigator blinded to the study allocation. The effect size will be measured using relative risk, absolute risk reduction, and numbers needed to treat.

**Table 1:** **Grading system for catheter tip location** [21] **with additional grades 1a/1b**

Grade	Accuracy of placement	Location of catheter tip
1	Optimal/adequate	Ipsilateral frontal horn, including tip of third ventricle: 1a, no contact with ventricle wall; 1b, contact with ventricle wall
2	Suboptimal in non-eloquent tissue	Contralateral frontal horn or lateral ventricle/corpus callosum/interhemispheric fissure
3	Suboptimal in eloquent tissue	Brainstem/cerebellum/internal capsule/basal ganglia/thalamus/occipital cortex/basal cisterns

#### Secondary outcome measures

The secondary outcome measures will be the frequency of placements required, the frequency of completed placements within the ventricle of the perforated part of the catheter tip, the frequency of very early and early shunt failures (revision of the ventricular drainage within 24 hours and within the hospital stay), and the frequency and percentage of complications (procedure-related and nonsurgical) at discharge. The effect size will be measured using relative risk, absolute risk reduction, and numbers needed to treat where possible.

### Data documentation

The following parameters will be recorded and used to demonstrate successful randomization and the presence of even baseline groups (Table 1):

1. Age (years);
2. Gender (nominal male/female);
3. Glasgow Coma Score on admission (ordinal, 1 to 15-point scale);
4. Cause of hydrocephalus/slit ventricles Preoperative ventricle size - width (millimeters);
5. Preoperative ventricle size - using the frontal occipital horn ratio (FOHR; millimeters);
6. Preoperative ventricle size - and the width of the lateral ventricle in the coronal plane between the medial wall of the corpus callosum and the septum;
7. Preoperative ventricle size - using the Evans ratio (bifrontal ventricular span, the ratio of maximum width of the frontal horns to the maximum width of the inner table of the cranium; millimeters),
8. Episode of care (nominal, *de novo*/revision) Time from symptom onset to *admissi*on (hours);
9. Time from symptom onset to randomization (hours);
10. Time from symptom onset to EVD (external ventricular drainage placement (hours);
11. Duration of hospital stay (days);
12. Duration of EVD being in place (days);
13. Presence of CSF infection during the first 14 days, as defined by modified clinical diagnostic criteria for device-associated meningitis (treatment required on either positive culture, or elevated cell count, red cell:white cell ratio, increased lactate, and/or decreased glucose (nominal, yes/no) [22].
14. The EVD catheter placed for ventriculoperitoneal shunting (nominal, yes/no).
15. Any associated endoscopic procedure, for example, third ventriculostomy (freetext);
16. Neurosurgical experience of primary operator (years).

### Consent procedure

Informed consent is obtained from each patient or legal representative. Patients capable of consenting will be informed about the study details themselves. The detailed explanation of the study to the patient or legal representative must be carried out using appropriate explanations and words depending on the previous medical knowledge of the respective person and his or her level of education. During the explanations, the respective person will be asked on a regular basis if they understand the conveyed information and if any questions have arisen. In addition to these verbal explanations the patient or legal representative will be given a leaflet containing the study details. After reading the leaflet the respective person will be given as much time as they demand for the decision on study participation. This study evaluates the outcome of an intervention performed often in an emergency situation. Since approximately half of patients will not be able to give informed consent at admission, the informed consent procedure for this study will be delayed in a so-called emergency procedure. If the patient is capable of giving informed consent and/or a family representative is available in due time, an independent neurosurgeon not involved in the patient’s treatment or in the trial may be asked for study approval. This option was introduced into the consent procedure because already available data on smart-phone guided ventriculostomy suggest a potentially beneficial effect of the measure for the patient. However, this was performed in a small group of 35 patients [20]. Therefore it shall not be categorically withheld from patients who are not capable of deciding whether to participate in the study or not and who do not have a legal representative. The ethical vote for this kind of procedure was obtained by the local ethics committee (approval number: CER 13–175, approved on 24 March 2014). As soon as a legal representative is available and/or the patient is capable of consenting to the study, he or she must be asked to give informed consent. If the patient or their legal representative refuses consent after inclusion by the advice of an independent physician, the patient’s further study participation is no longer possible. In this case, however, the patient or their legal representative is asked to give consent for evaluation of already acquired data.

### Sample size analysis

Sample size analysis estimates (hit and miss rates) were based on the results of observational studies. In a recent study of our group on the accuracy of ventricular catheter placement, using anatomic landmarks versus neuronavigation or XperCT-laser guidance, ventricles were hit in 69.2% of catheter insertions (31% miss rate) using anatomical landmarks (non-assisted), as compared to 82.3% with the use of guidance (neuronavigation or XperCT-guidance, *P* = 0.043) [23]. The placements that were non-assisted were significantly more likely to be extraventricular than those using guidance (odds ratio: 3.73; 95% confidence interval: 1.24 to 11.19; *P* = 0.019). Given data from other retrospective studies on ventricular catheters (in the context of ventriculoperitoneal shunts), similar rates of between 25 and 45% [4–7] malpositioning are described.

To detect a relative risk of 0.5, which is an absolute risk reduction of 15.5%, 118 patients in each of the two study arms are needed to gain a power of 80%, using a two-sided significance level of 95%. The final planned study size is to include and randomize 236 patients [24].

### Data management

Data specified in the trial protocol will be documented in the patient’s digital records. Additional clinical data are available from the electronic patient files. The investigating physician is responsible for appropriate completion of the form. The authors (AS and NS) are responsible for database development, data acquisition, data storage, and validation. Data validation includes controls of completeness, consistency, and plausibility of the data documented in the case report form (CRF) = case report formand electronic dossier using a query system between data management and the investigating physician.

### Adverse events and severe adverse events

The term adverse event (AE) describes any sign, symptom, syndrome, or any disease occurring newly in a trial participant after consent to the trial and being of particular interest for the assessment of the disease or the security of the therapeutic concept. Any AE is documented in the CRF or electronic file of the patient.

For the purposes of this trial the definition of an adverse event will consist of any one of the following:

1. Impairment of the general condition of the patient,
2. Physical injury, including falls,
3. Infection other than wound infection, sepsis or Systemic Inflammatory Response Syndrome (SIRS) Intracranial bleeding in the proximity of the ventricular catheter,
4. Wound infection or dehiscence at the emersion site of the ventricular catheter,
5. Newly occurred neurological deficit,
6. Arterial or venous thrombosis,
7. Any suspicious findings that may have relationship to the study.

For the purposes of this study the definition of a serious adverse event (SAE) will consist of any one of the following:

1. Fatal events or death,
2. Any life-threatening condition,
3. Disabling or incapacitating events,
4. Events that require prolongation of hospitalization,
5. Events that require intervention to prevent permanent impairment.

The relation of the AE or SAE (as a cause or result) to any other AE or SAE will be scored on a two-point scale; 0 - no, 1 - possible (brief description), or 2 - yes (give brief description). The investigator’s estimation of the severity of the problem to the study or study device (to be measured only, if the AE or SAE is or might be related to study) will be measured and scored on a three-point scale; 1 - mild, 2 - severe, or 3 – life-threatening. The investigator’s recommendation to change the protocol, informed consent, or assent form will be given as a binary answer of yes (with a brief description) or no. The outcome of AE or SAE will be assessed until discharge using a four-point scale; 1 - full recovery, 2 - permanent deficit, 3 - not yet fully recovered, or 4 - not known.

### Reporting and statistical analysis

The reporting of this trial will conform to the reporting guidelines outlined in the Consolidated Standards of Reporting Trials (CONSORT) for non-pharmacological interventions statement of 2010 [25]. The statistical analysis will be by the intention-to-treat principle. The occurrence of the primary outcome (number and frequency of correct ventriculostomy) will be compared between the two randomization groups. The secondary outcome analyses will compare the above-described variables between randomization groups. Subgroup analyses will be performed to evaluate whether other parameters such as age, ventricle size, diagnosis, or experience of the surgeon may influence the results. Data are presented as mean and standard deviation or as median with 25/75 percentiles. Stata version 11.2 StataCorp. 2013. *Stata Statistical Software*, College Station, Texas, United States) will be used for all analyses.

### Interim analyses

This trial will follow a group sequential design, with two stop-points (interim and final analysis; K = 2). The trial will stop if efficacy rules are met. The endpoints assessed at the first stop-point will be based on primary outcomes and adverse events. The first will be the interim analysis which will occur after the accrual of 60 patients in each arm (120 total), thus representing an information fraction of 0.51 at interim analyses. For the efficacy stop-point the commonly used O’Brien-Fleming approach will be used, and a *P* value of 0.0051 at the interim and 0.0415 at the final analysis will be used [26].

## Discussion

Studies assessing catheter malplacement find a high percentage of cases of misplacement. Following head trauma, mass lesions, or in slit ventricles, there is a situation of altered anatomy, rendering catheter placement difficult. For example, the ventricles may be compressed and deviated and therefore difficult to cannulate. Furthermore, if the ventricular catheter is placed in the context of a shunt, high revision rates add to patient morbidity and procedural costs. As there is a need to improve current practices in the placement of ventricular catheters, this VENTRI-GUIDE protocol aims to assess if a portable, easy-to-use, mini-tablet-assisted guide for ventriculostomy can increase the accuracy of ventriculostomy in comparison with the freehand pass technique. Our primary outcome is the rate of correct placements of the ventricular catheter, measured using a three-point grading scale for the outcome of each ventriculostomy.

### Factors influencing ventricular catheter placement

There are anatomical, technical, and individual patient- and surgeon-related aspects to consider when placing a ventricular catheter, in particular, ventricular size and choroid plexus distance. Preoperative ventricle size is significant in its relation to the accuracy of catheter placement [1], which is most relevant in pediatric patients [27]. Larger ventricles are more likely to have optimally placed catheters, which decreases the risk of proximal obstruction, and hence increases shunt survival. Interestingly, a recent study by Wilson *et al*. in an adult study population (170 patients, mean age 56 years), which is probably comparable to our supposed study group, did not show a correlation of ventricular size by freehand technique (measured as bifrontal ventricular span or by the Evans ratio) with risk of inaccurate placement [7]. Also the distance to the choroid plexus is described as a factor contributing to catheter occlusion. Ventricular size and choroid plexus distance are related to the first episode of shunt failure, particularly in the pediatric population, because the ventricle size is smaller and the choroid plexus is larger in the pediatric population [28].

Obstruction, the main cause for shunt failure, may be due to blockage by the choroid plexus, ventricular debris or glial tissue growing into its lumen, or due to reduction in size of the drained ventricle. A retrospective series considering 90 adult and pediatric patients with 116 shunt failures identified as main cause for shunt failure proximal catheter obstruction (38%), followed by infection (29%) [6]. Mechanical obstruction can be avoided by inserting a sufficiently long catheter with its tip just anterior to the foramen of Monro [12]. The length, determined in a previous study from the CT scan scout film of the patient, was half of the distance between the external auditory meatus and the midpoint of the coronal suture of the skull, studied in 175 patients, with good results [12]. Also, Wan *et al*. identified catheter length as a relevant factor in avoiding obstruction. In contrast to their expectations they found, after correcting for age and approach, that a larger catheter (greater than 8.5 cm) was better placed than a shorter catheter [1]. Finally, the cause of the hydrocephalus is related to the survival of the ventricular catheter, with a higher risk in hemorrhagic ventricles or with ventriculitis. Gender, American Society of Anesthesiologists (ASA) Physical Status classification class, emergency, use of prophylactic antibiotic agents, and duration of surgery were not related to shunt failures in a pediatric population with 90% use of a ventriculo-peritoneal shunt [5].

### Surgical technique

The classical freehand technique uses anatomical landmarks such as the inner canthus and external auditory meatus. The catheter is placed in the anterior horn of the lateral ventricle through a burr hole just anterior to the coronal suture in the mid-pupillary line (approximately 11 cm above the nasion and 3 cm from the midline). It is associated with difficulty in gaining optimum insertion angle and catheter length with better success rates in experienced surgeons. Optimal trajectories for a frontal approach were in the mean 42° in the sagittal plane and 30° in the coronal plane [5].

The surgical technique using the frontal burr hole as in the present study has the shortest intracerebral route, with more consistent anatomical landmarks [1]. Others prefer the parietal approach, as the atrium is usually the most dilated part of the lateral ventricle [5], proposed for patients with expected relevant reduction of ventricular size after shunting [29]. The frontal and parietal burr holes are associated with better placement compared to occipital burr holes [1]. In the present protocol, a uniform frontal approach has been chosen.

For difficult implantation situations and/or inexperienced neurosurgeons, other techniques have been described to reduce the rate of malplacements and related complications. Frameless neuronavigational stereotaxy [4], conventional stereotactic, and robotic-image guidance techniques [30] (n = 17 patients) used for small or slit ventricles have demonstrated accurate placement of ventricular catheters, however, in relatively small patient groups. Intracranial neuroendoscopy is another alternative in selected cases [17]. Also real-time ultrasound-assisted catheter placement is used in older children, with the disadvantage of needing a larger burr hole of 1.5 to 2 cm [15]. There are some disadvantages of these sophisticated methods, such as the prolongation of operative time and dependence on expensive, and sometimes complicated technologies [18, 31]. Nevertheless, the complication rate might be reduced. In a recent study by our group on the accuracy of ventricular catheter placement using anatomic landmarks versus neuronavigation or XperCT-laser guidance, ventricles were hit in 69.2% of catheter insertions using anatomical landmarks (non-assisted), as compared to 82.3% with the use of guidance (neuronavigation or XperCT guidance, *P* = 0.043) [23]. The placements that were non-assisted were significantly more likely to be extraventricular than those using XperCT (odds ratio: 3.73; 95% confidence interval: 1.24 to 11.19; *P* = 0.019). Attempts with non-assisted placement were more likely to result in an unsafe trajectory compared to those using neuronavigation (odds ratio: 3.16; 95% confidence interval: 1.27 to 7.84; *P* = 0.013). However, there was no difference in final intraventricular placement comparing XperCT guidance placement compared to the neuronavigation placement method (odds ratio: 1.44; 95% confidence interval: 0.55 to 3.78; *P* = 0.46).

### Limitations

Simply monitoring of patients and tracking within a study will already improve performance in most cases, and attention to catheter placement will likely have a similar effect [19]. In addition, this trial is structured to detect a rather large difference in ventricular misplacements. We are seeking an approximate 15% change, from 35 to 20% hit rate, something which may be very difficult to achieve.

A key limitation is the expected to be an imbalance of surgical skill and/or a learning curve. The surgeons who use the freehand technique will be more experienced using the freehand technique than the mini-tablet-assisted method because they will have performed more procedures using freehand, and we may underestimate the effect size of the guide-assisted method.

In addition, we feel that a learning curve is present, but small because of the similarity between the two procedures. Nonetheless, this limitation is common to surgical randomized controlled trials and will be minimized as best as possible within the limitations of our resources, mostly by doing frequent teaching sessions prior to the opening of the trial. The clinical diagnosis might have an impact on the rate of misplacements with highest rates for suboptimal placements in trauma patients with a midline shift on a CT scan [21], an effect reducible using randomization, as in the present study.

In conclusion, we have developed a protocol to evaluate if an easy-to-use, portable, image-guided system could reduce the need for multiple passes and improve the rate of accurate catheter placement compared to the standard freehand technique. In contrast with earlier studies, this study is prospective, controlled, and randomized. Considering the high revision rates for patients, especially with small ventricular sizes, the guided technique may lead to better outcome and may be cost-effective. Since the outcome of catheter placement is assessed using an established grading system for catheter tip location post-surgery as the primary endpoint, this study will provide an answer for whether an increase of accuracy for ventriculostomy can be reached in patients with guided ventriculostomy.

### Roles and responsibilities

#### Principal investigator and executive team

Asita Sarrafzadeh (AS) is the principal investigator and will coordinate the trial, be responsible for preparation of the protocol and questionnaires, day-to-day conduct of the trial, medical support in day-to-day conduct of the trial, evaluating the cause of an unfavorable outcome, supplying information about the trial, day-to-day conduct of the trial database, and the writing and publication of the results. Nicolas Smoll (NS) is responsible for preparation of the protocol and questionnaires, statistical validity, database construction, data analysis, the Data and Safety Monitoring Board, and the writing and publication of the results. Karl Schaller (KS) is responsible for medical support in the day-to-day conduct of the trial, financing, and publication of the results. The Data and Safety Monitoring Board will consist of AS and NS.

## Trial status

The planned study start date is 1 March 2015. The estimated study duration will be three years.

## Abbreviations

CT: Computed tomography.
